# Identification and Characterization of Novel Genotoxic Stress-Inducible Nuclear Long Noncoding RNAs in Mammalian Cells

**DOI:** 10.1371/journal.pone.0034949

**Published:** 2012-04-19

**Authors:** Rena Mizutani, Ai Wakamatsu, Noriyuki Tanaka, Hiroshi Yoshida, Naobumi Tochigi, Yoshio Suzuki, Tadahiro Oonishi, Hidenori Tani, Keiko Tano, Kenichi Ijiri, Takao Isogai, Nobuyoshi Akimitsu

**Affiliations:** 1 Radioisotope Center, The University of Tokyo, Tokyo, Japan; 2 Graduate School of Pharmaceutical Sciences, The University of Tokyo, Tokyo, Japan; 3 Asahi General Hospital, Chiba, Japan; 4 Medical Consultation Office, Incorporated, Yokohama, Japan; Sun Yat-sen University Medical School, China

## Abstract

Whole transcriptome analyses have revealed a large number of novel transcripts including long and short noncoding RNAs (ncRNAs). Currently, there is great interest in characterizing the functions of the different classes of ncRNAs and their relevance to cellular processes. In particular, nuclear long ncRNAs may be involved in controlling various aspects of biological regulation, such as stress responses. By a combination of bioinformatic and experimental approaches, we identified 25 novel nuclear long ncRNAs from 6,088,565 full-length human cDNA sequences. Some nuclear long ncRNAs were conserved among vertebrates, whereas others were found only among primates. Expression profiling of the nuclear long ncRNAs in human tissues revealed that most were expressed ubiquitously. A subset of the identified nuclear long ncRNAs was induced by the genotoxic agents mitomycin C or doxorubicin, in HeLa Tet-off cells. There were no commonly altered nuclear long ncRNAs between mitomycin C- and doxorubicin-treated cells. These results suggest that distinct sets of nuclear long ncRNAs play roles in cellular defense mechanisms against specific genotoxic agents, and that particular long ncRNAs have the potential to be surrogate indicators of a specific cell stress.

## Introduction

One of the greatest surprises of the human genome project has been that the extent of non-coding genomic regions increases markedly with developmental complexity. This is in contrast to protein-coding regions [1], [2]. Whole transcriptome analyses utilizing high-density tiling microarrays and deep sequencing have revealed that a huge number of novel transcripts with low protein-coding potential (named noncoding RNAs (ncRNAs)) are transcribed from these non-coding genomic regions [3]–[6]. This discovery has opened new research avenues, with the aims of revealing the functions of ncRNAs, towards understanding complex biological systems in higher organisms. These ncRNAs can be roughly classified into two groups based on their length: short transcripts (20–200 nucleotides), such as microRNAs (miRNAs) and piwi-interacting RNAs; and long transcripts (>200 nucleotides) [7]. Although the biological importance of short ncRNAs has been documented in recent years, the physiological functions of long ncRNAs are poorly understood.

Recently, several studies have reported that nuclear long ncRNAs play pivotal roles in mammalian cells, including transcriptional regulation, regulation of splicing, and epigenetic regulation. A key player in dosage compensation of the mammalian X-chromosome, *XIST*, is distributed along the target X-chromosome, where it silences gene expression by changing chromatin structure [8]–[10]. *NEAT1* (also known as MEN epsilon/beta) localizes to nuclear paraspeckles, where it acts as an essential component of the paraspeckle structure [11]–[14]. *MALAT1* also localizes to nuclear speckles, and is involved in transcriptional and post-transcriptional gene expression [15], [16]. *ANRIL* recruits polycomb repression complex 2 (PRC2) to the *INK4A*-*ARF*-*INK4B* gene cluster and is involved in the silencing of the *INK4A* and *INK4B* genes [17]–[19]. *Kcnq1ot1*, which is involved in bidirectional silencing of genes in the Kcnq1 domain, interacts with the histone methyltransferase G9a and the PRC2 complex [20]. *HOTAIR* works as a molecular scaffold to regulate histone modification through its interaction with two distinct histone modification complexes: PRC2 and LSD1/CoREST/REST [21], [22]. This accumulating evidence raises the intriguing possibility that nuclear long ncRNAs play important roles in controlling various aspects of biological function in the nucleus.

The stress response is a highly conserved cellular response to environmental changes with transient reprogramming of transcriptional, translational, and post-translational activities [23]. Depending on the severity and duration of the stress encountered, cells either re-establish cellular homeostasis to the former state or adopt an altered state in the new environment. Expression of ncRNAs as well as mRNAs is regulated by stress and environmental stimuli, and a distinct set of ncRNAs accumulates in stimulated cells, suggesting that ncRNAs are important and tightly controlled in response to stress and environmental stimuli [7], [24]. The *SatIII* ncRNAs, which are transcribed from the satellite III repeat sequence that is present mainly in the pericentromeric region of human chromosome 9, are dependent on heat shock transcription factor HSF1. The *SatIII* ncRNAs form nuclear stress bodies and play an important role in the heat shock response [25], [26]. The endogenous *BACE1*-antisense transcript, which is the beta-site cleaving enzyme essential for the biosynthesis of amyloid beta 1–42 and 1–40, is induced by various cell stressors [27]. Numerous large intergenic ncRNAs (lincRNAs) are regulated by the p53 pathway involved in the DNA damage response [28]. The expression level of the RNA gene *PRINS* is increased by stress signals such as ultraviolet-B irradiation, viral infection, and translational inhibition [29]. Although several ncRNAs have been reported to be the stress-inducible transcripts, the complete picture of stress-inducible nuclear long ncRNAs remains largely unknown.

In this study, we sought to identify novel mammalian nuclear long ncRNAs that are involved in the genotoxic stress response. We selected ncRNA candidates from human ESTs and full-length cDNA sequences that are available in public databases (NCBI Reference Sequences and Ensembl human gene transcripts), using the widely accepted ncRNA criteria: the predicted open reading frame should be less than 300 nucleotides and any Kozak sequence around the first ATG (expected initiation codon) should be of low potential. Then, we determined their cellular localization in HeLa Tet-off (TO) cells to identify nuclear long ncRNAs. Finally, we identified 25 novel nuclear long ncRNAs and investigated their responses to genotoxic stress.

**Figure 1:**
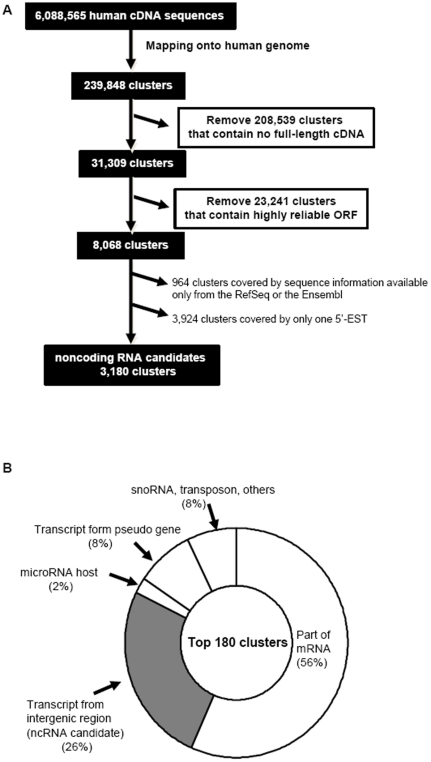
Procedure for the identification of ncRNA candidates. (A) Outline of our gene-prediction method from human full-length cDNAs and ESTs mapped to the human genome. ORF, open reading frame. (B) Classification of 180 ncRNA candidates analyzed manually using the UCSC genome browser.

## Materials and Methods

### Cell culture, RNA interference, and drug treatment

HeLa TO cells (Clontech) and MCF-7 (kindly gifted by Dr. Kohtake) were grown in Dulbecco's modified Eagle's medium supplemented with 10% fetal bovine serum and antibiotics at 37°C and 5% CO_2_ in a humidified incubator.

The sequence of the small interfering RNA (siRNA) targeting *UPF1* was as follows: 5′-GAU GCA GUU CCG CUC CAU UdT dT-3′; the sequence of the control siRNA was 5′-GTA CCT GAC TAG TCG CAG AAG-3′. The siRNAs were transfected into cells using Lipofectamine RNAiMAX (Invitrogen) according to the manufacturer's instructions. The siRNA duplexes were used at a final concentration of 10 nM, and the cells were harvested 48 h after transfection.

To induce genotoxic stress, HeLa TO cells (2×10^5^ cells in a 12-well plate) were treated with mitomycin C (MMC) at final concentrations of 5, 10, or 20 µg/ml, or with doxorubicin (DOX) at final concentrations of 0.5 or 1.0 µM. We harvested cells 6 or 8 h after treatment.

### Selection of ncRNA candidates from databases

The 5′- and 3′-ends of cDNA sequences and the full-length cDNA sequences [110,000 full-length human cDNA sequences available from public databases, 30,000 from human RefSeq (NCBI Reference Sequences, human, ver. 2005.10.17; http://www.ncbi.nlm.nih.gov/RefSeq/), and 48,000 from Ensembl human gene transcripts (human-38.36g/UCSC hg18; http://www.ensembl.org/index.html)] were mapped onto the human genome (UCSC hg 18 NCBI Build 36.1) and clustered. The number of clusters was 239,848. The information is available in the FLJ Human cDNA Database ver. 3.0 (http://flj.lifesciencedb.jp) [30]. We removed 208,539 clusters that consisted of expressed sequence tags (ESTs) only. By following the widely accepted mRNA criteria – that the predicted open reading frame should be larger than 300 nucleotides (100 amino acids) and the Kozak sequence around the first ATG (expected initial codon) should be of high potential – the 23,241 clusters predicted to encode protein-coding mRNAs were removed [30]. To improve reliability, we also removed the 4,888 clusters for which the gene locus was covered by sequence information from Ensembl only or NCBI only, or by just one 5′-EST.

### Research ethics

This study was conducted according to the principles expressed in the Declaration of Helsinki. The human tissue samples were prepared at the Asahi General Hospital. The details of the subjects were as follows: Case 1 (age: 30 years; post-mortem interval: 3 hours; cause of death: malignant lymphoma; the treatment: combination chemotherapy with rituximab and cytotoxic agents including doxirubicin) and Case 2 (age: 67 years; post-mortem interval: 2 hours; cause of death: pulmonary actinomycosis; the treatment: ampicillin and sulbactam). The Asahi General Hospital obtained informed consent from all subjects. The Asahi General Hospital Institutional Review Board and the University of Tokyo Institutional Review Board approved the use of the human tissue in this study according to the Ethical Guidelines of the Ministry of Health, Labour, and Welfare of Japan. Written informed consent for study participation was obtained from all participants and was recorded by the physician on a study-participation sheet. The data were analyzed anonymously.

### Quantitative real-time RT-PCR (qRT-PCR)

Total RNA was extracted from tissues or cells with RNAiso Plus (TaKaRa) according to the manufacturer's instructions. The isolated RNA was reverse transcribed into cDNA using PrimeScript RT Master Mix (Perfect Real Time) (TaKaRa). All cDNA was amplified using the primer sets listed in Table S1. Glyceraldehyde 3-phosphate dehydrogenase (*GAPDH*) was used as a reference gene for normalization. SYBR Premix Ex Taq II (Perfect Real Time) (TaKaRa) was used for PCR according to the manufacturer's instructions. Quantitative real-time RT-PCR was performed using a Thermal Cycler Dice Real Time System (TaKaRa).

### Northern blot hybridization

Total RNA (10 µg) was separated on a 1.1% (w/v) agarose gel containing 0.08% formaldehyde and transferred to a positively charged nylon membrane (Millipore). After UV cross-linking, blots were hybridized to ^32^P-labeled riboprobes at 52°C, overnight, in Ultrasensitive Hybridization Buffer (Applied Biosystems). An autoradiographic image was captured and quantified using a FLA9000 biomolecular imager (FUJIFILM).

### Cell fractionation

HeLa TO cells (approximately 1×10^7^ cells) were collected using a rubber policeman and centrifuged at 500× *g* for 5 min. The cell pellet was washed in ice-cold RSB150 buffer [10 mM Tris-HCl pH 7.4, 150 mM NaCl, 2.5 mM MgCl_2_] and centrifuged as before. Then, the cell pellet was resuspended in 800 µL of ice-cold RSB150 buffer. The cells were divided into two tubes for the total or nuclear/cytoplasmic fractions. For the nuclear/cytoplasmic fractions, 0.25 mg/mL digitonin was added to the cells and incubated for 5 min on ice. The cells were centrifuged at 3,000× *g* for 1 min at 4°C, yielding the cytoplasmic (supernatant) and nuclear (pellet) fractions. The supernatant was kept on ice. The pellet was washed twice in ice-cold RSB150 buffer. Then, the cell pellet was resuspended in 400 µL of ice-cold RSB150 buffer and 0.5% Triton X-100. RNA was extracted from the obtained fractions using ISOGEN LS (Nippon Gene) according to the manufacturer's instructions.

**Figure 2:**
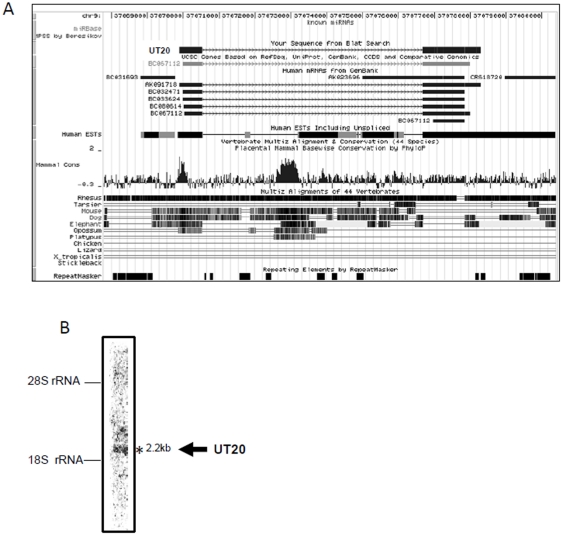
Examples of ncRNA candidates. (A, C) UCSC genome browser analysis of UT20 (A) and UT21 (C). (B, D) Northern blot analysis of UT20 (B) and UT21 (D). Total RNA from HeLa Tet-off cells was analyzed. The positions of the ribosomal RNAs (rRNAs) are shown to the left of the blots. Asterisks indicate bands corresponding to the expected size.

## Results

### Identification of novel nuclear long ncRNAs in mammalian cells

The initial selection procedure is summarized in Figure 1A. To construct the initial dataset for the identification of novel long ncRNAs, we obtained 6,088,565 human transcript sequences as described in a previous report [30]. We obtained 31,309 clusters by mapping all sequence data onto the human genome followed by clustering, Intris analysis and selection of clusters that all contained a full-length cDNA, according to a previous report [30]. We excluded the 23,241 clusters that were predicted to be protein-coding mRNAs, and also removed the 4,888 clusters for which the gene locus was covered by sequence information available only from the RefSeq or the Ensembl, or by just one 5′-EST. Ultimately, we obtained 3,180 clusters as candidates likely to encode ncRNAs.

We selected the top 180 clusters containing a high number of cDNA sequences as the clusters with supposed high expression (Table S2). By analysis of gene structure using the UCSC genome browser, we manually removed the clusters that contained or overlapped with annotated pseudogenes, transposons, or protein-encoding genes. Thus, 46 clusters were selected as transcriptional units likely to be ncRNAs; these were designated UT1–46 (Figure 1B, Table 1). We determined that 36 of the 46 ncRNA candidates were expressed in HeLa TO cells (data not shown) by qRT-PCR analysis. All 36 candidates were longer than 200 nucleotides; that is, they fulfilled the established criteria for long ncRNAs. To verify their existence, we performed northern blotting for two candidates (UT20 and UT21), and detected signals corresponding to the expected size (Figure 2). Secondary structure prediction revealed that both UT20 and UT21 form a complex structure (Figure S1). Next, we investigated sequence conservation using the UCSC genome browser. The majority of these ncRNA candidates displayed a low level of sequence conservation across mammalian species. The level of sequence conservation for several ncRNA candidates (UT16, UT18, UT19, UT22, UT31, UT26, UT30, UT32, UT36, UT45, and UT46) was high (Figure S2 and data not shown).

**Table 1:** Novel noncoding RNA candidates.

Code name	Representative sequence name	Code name	Representative sequence name
UT1	AK127256	UT24	AK123363
UT2	BX537518.1	UT25	AK090827
UT3	BX648321.1	UT26	AL832786.1
UT4	BX648343.1	UT27	AK022839
UT5	AL713664.1	UT28	AL049256
UT6	AL833456.1	UT29	AK057867
UT7	BX641108.1	UT30	BX648070.1
UT8	BX641108.1	UT31	AK094705
UT9	NR_015389	UT32	AK126393
UT10	AL117555.1	UT33	BX538228.1
UT11	AK055564	UT34	NR_015411.1
UT12	AK092215	UT35	AK054990
UT13	NR_027183.1	UT36	NR_024281.1
UT14	AL157455.1	UT37	NR_015439.1
UT15	AB007979.1	UT38	AK057104
UT16	BC041380.1	UT39	AK021734.1
UT17	AL049354.1	UT40	AK055559
UT18	AL049423.1	UT41	BX647964.1
UT19	AK092810	UT42	AL359605.1
UT20	AK091718	UT43	BX537481.1
UT21	AK055657	UT44	AK091396.1
UT22	AK021884	UT45	BX648677.1
UT23	NR_003367	UT46	AK093733

**Figure 3:**
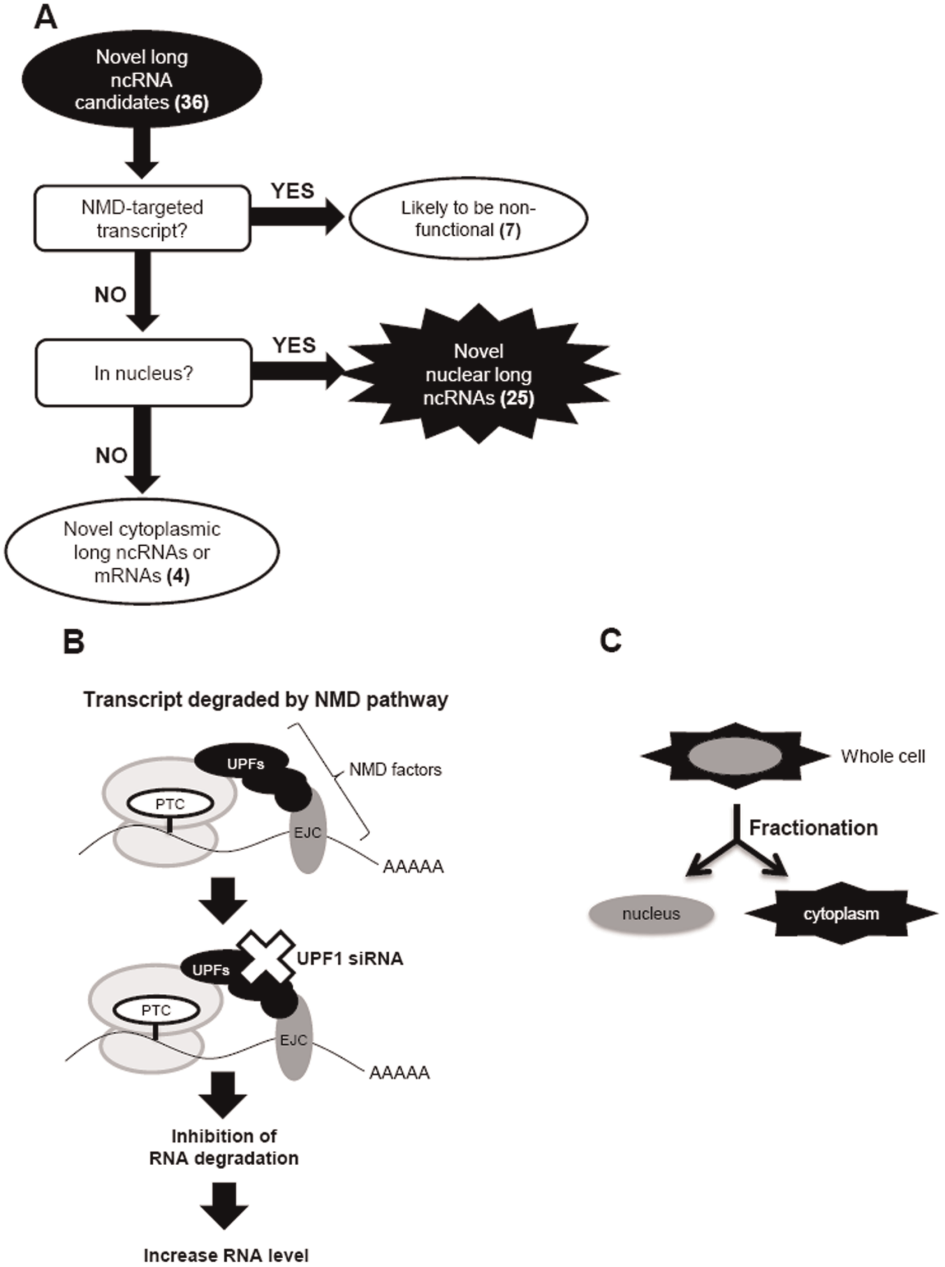
Strategy for the selection of ncRNAs. (A) A schematic of the selection procedure for ncRNAs. They were selected based on their lack of susceptibility to nonsense-mediated RNA decay (NMD) and nuclear localization. The identities of the transcripts in each category are given in Table 1. (B) Schematic of aberrant mRNAs harboring a premature termination codon (PTC) and surveillance complexes containing UPF factors. EJC indicates an exon junction complex, which is an essential component for NMD in mammalian cells. (C) Details of the sub-cellular localization analysis of transcripts.

**Figure 4:**
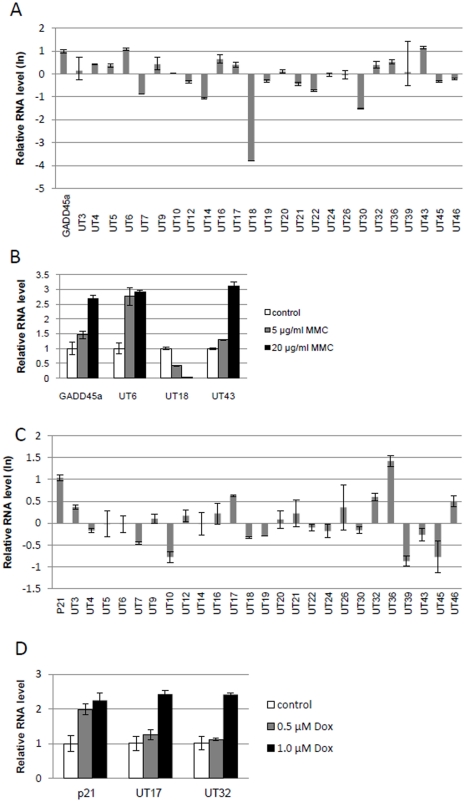
Alteration of nuclear long ncRNA expression by anticancer agents in HeLa Tet-off (TO) cells. (A, B) HeLa Tet-off (TO) cells were treated with 20 µg/ml MMC (A), and 5 or 20 µg/ml MMC (B) and subjected to qRT-PCR. RNA levels were normalized to those of *β-actin*, and are presented relative to non-treated cells. *GADD45a* was used as a positive control induced by MMC. (C, D) HeLa Tet-off (TO) cells were treated with 1.0 nM DOX (C) and 0.5 or 1.0 nM DOX (D) and subjected to qRT-PCR. RNA levels were normalized to those of *GAPDH*, and are presented relative to non-treated cells (con). *p21* was used as a positive control induced by DOX. Error bars show the experimental error of two experiments.

**Figure 5:**
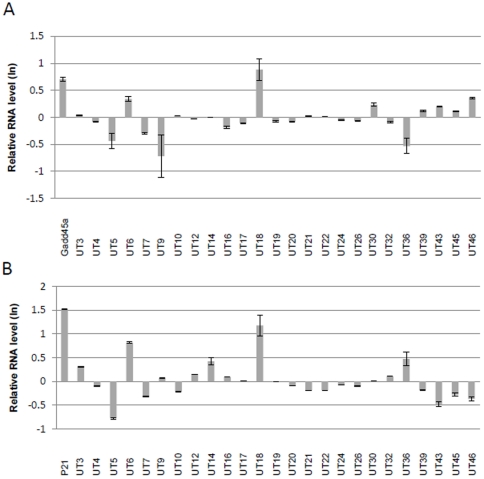
Alteration of nuclear long ncRNA expression by anticancer agents in MCF-7 cells. (A) MCF-7 cells were treated with 20 µg/ml MMC and subjected to qRT-PCR. RNA levels were normalized to those of *β-actin*, and are presented relative to non-treated cells (con). *GADD45a* (induced by MMC) was used as a positive control. (B) MCF-7 cells were treated with 0.5 nM DOX and subjected to qRT-PCR. RNA levels were normalized to those of *β-actin*, and are presented relative to non-treated cells (con). *P21* (induced by DOX) was used as a positive control. Error bars show the experimental error of two experiments.

**Figure 6:**
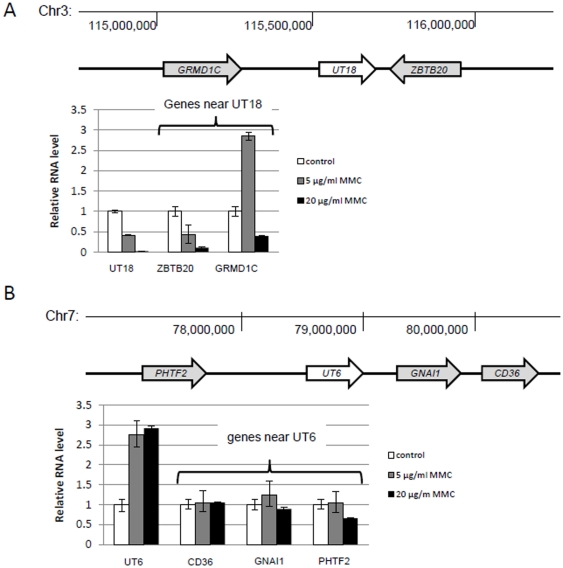
Expression of the genes neighboring stress-inducible nuclear long ncRNAs under MMC treatment. HeLa Tet-off (TO) cells were treated with MMC and subjected to qRT-PCR. (A) UT18-neighboring genes were co-regulated, whereas (B) UT6-neighboring genes were not. Error bars show the experimental error of two experiments.

To select the transcripts likely to be *bona fide* functional ncRNAs, we analyzed them further (Figure 3A). First, we excluded the ncRNA candidates that were likely to be degraded through nonsense-mediated RNA decay (NMD). NMD is a mechanism for eliminating aberrant mRNAs harboring premature termination codons or genomic noise such as inactive transposons and pseudogenes [31], [32]. Based on the concept that NMD eliminates unnecessary transcripts, we assumed that NMD-targeted transcripts would likely be a non-functional transcript. To identify the NMD-targeted transcripts, we analyzed the expression levels of the ncRNA candidates in cells eliminating UPF1, an essential NMD factor (Figure 3B). The levels of known NMD-targeted ncRNAs *UHG* and *GAS5* were increased 258 and 404%, respectively, in UPF1-knockdown cells compared with those in control cells. Therefore, we deemed the candidate ncRNAs to be targeted by NMD when the transcript levels exceeded 200% of control levels in these cells. Seven out of the 36 candidates were judged to be NMD-targeted transcripts (Table S3) and were excluded from further analysis.

We then determined the sub-cellular distribution of the remaining 29 candidates, using cytoplasmic and nuclear cell fractions (Figure 3C). We established the nuclear/cytoplasmic (N/C) ratio using two control transcripts, *MALAT1* and *GAPDH*, known to be localized in the nucleus and cytoplasm, respectively. The N/C ratios of *MALAT1* and *GAPDH* were 10.6 and 0.5, respectively. Therefore, we defined the subcellular localization of the transcripts as: N/C ratio >2, nuclear localization; N/C ratio 0.5–2, nuclear and cytoplasmic localization; and N/C ratio <0.5, cytoplasmic localization. Among the 29 candidates, 25 were localized in the nucleus (Table S4). As nucleus-localized RNAs have a low potential to be translated, we judged that these were the most likely to be *bona fide* ncRNAs. The frequency of nuclear long ncRNAs (25/29, approximately 86%) in this study is higher than the frequency described in the previous report, in which approximately half the intergenic non-coding transcripts were retained in the nucleus [33].

### Expression profiles of nuclear long ncRNAs in human tissues

It has been reported that a subset of long ncRNAs is expressed in a tissue-specific manner [34], [35]. To examine whether the 25 nuclear long ncRNAs described here were expressed in a tissue-specific manner, we determined the distribution and relative abundance of these long ncRNAs using qRT-PCR in seven human tissues: cerebrum (cortex), cerebellum (cortex), stomach (mucosa), pancreas, thyroid gland, lung, and heart (myocardium). The expression patterns were determined using RNAs from two individuals, with some exceptions. Their profiling data are shown in Figure S3. In contrast to a previous report [34], most long ncRNAs were expressed in all seven tissues, suggesting that they are ubiquitously expressed. Only UT36 was expressed in a subset of the tissues (Figure S3). In addition, we confirmed the expression of these long ncRNAs using a publicly available RNA Seq data set obtained from Illumina's Human Body Map 2.0 (Figure S4).

### Stress-induced expression of nuclear long ncRNAs

To investigate the functions of the 25 novel long ncRNAs, we determined the alteration in their expression level following treatment of HeLa TO cells with the DNA damaging agents MMC or DOX. Upon treatment with 20 µg/ml MMC or 1.0 µM DOX, the expression level of several long ncRNAs was altered. Moreover, the expression level of UT6, UT43, and UT18 was altered at each concentration of MMC4A, B, and that of UT17, UT32, UT36 and UT46 was altered at each concentration of DOX (Figures 4C and 4D). These data suggest that these long ncRNAs are involved in the cellular responses to DNA damaging agents. We also evaluated the altered expression of long ncRNAs in MCF-7 cells treated with 20 µg/ml MMC or 0.5 µM DOX. MMC induced UT18 (Figure 5A), and DOX induced UT6 and UT18 (Figure 5B). These results suggest that the majority of anti-cancer agent-mediated alterations of long ncRNA expression were dependent on cell type.

It has been reported that rapid induction of immediate-early genes in response to stimulation is accompanied by co-upregulation of their neighboring genes [36]. The transcription of immediate early genes propagates outside the boundaries of the initial target genes and into transcribed genes up to 100 kb downstream. To investigate whether nuclear long ncRNAs were co-regulated with their neighboring genes in response to genotoxic agents, we analyzed the expression level of the ncRNA-adjacent genes in cells treated with MMC. UT18 and its neighboring genes, *GRMD1C* and *ZBTB20*, were simultaneously downregulated in cells treated with 20 µg/ml MMC (Figure 6A). UT6 was upregulated by MMC but the expression of its neighboring genes *CD36*, *GNAI1*, and *PHTF* was not altered (Figure 6B). These data suggest that several long ncRNAs specifically respond to genotoxic agents and as such, these long ncRNAs are least likely to be transcriptional noise.

## Discussion

Many studies have described that miRNA expression patterns are altered in response to stress and environmental stimuli [37]. However, there are few examples of long ncRNAs whose expression is altered by stress or other stimuli. In this report, we identified 25 novel nuclear long ncRNAs and demonstrated that the expression of a subset is induced by genotoxic agents. We did not find commonly altered nuclear long ncRNAs between MMC and DOX treatment. Although both drugs cause genotoxic effects [38], their mechanisms of action are different. DOX induces its genotoxic effect through intercalation into genomic DNA and/or inhibition of topoisomerase II [39]. MMC cross-links double-stranded DNA, consequently inhibiting DNA replication; it induces double-strand breaks [40]. Our data suggest that distinct sets of nuclear long ncRNAs play roles in cellular defense mechanisms against individual genotoxic agents. Our results also suggest that subset of long ncRNAs have the potential to be surrogate indicators of stress induced by specific genotoxic agents.

The nuclear long ncRNAs identified in this study are different from previously identified groups of ncRNAs, such as TSSas (transcription start site-associated RNAs) [41], PASRs (promoter-associated short RNAs), PALRs (promoter-associated long RNAs), or TASRs (termini-associated short RNAs) [42], because the ncRNAs described in this study do not flank the active promoters or terminators of previously annotated genes. Because our novel nuclear long ncRNAs are clearly detectable without depleting the nuclear RNA degradation pathway, they are different from CUTs (cryptic unstable transcripts) and PROMPTs (promoter upstream transcripts) [43], [44], which are only detectable in the absence of the nuclear RNA degradation pathway. A set of lincRNAs is regulated by p53, suggesting that some may be involved in the p53-mediated stress response. Sequence alignment revealed that the long nuclear ncRNAs identified in this study are distinct from reported lincRNAs (data not shown). We also determined that the novel long ncRNAs identified in this study do not overlap with previously collected long ncRNAs [45]–[47]. Moreover, measurement of the amount of RNA by next-generation sequencing suggested that the expression levels of most of the long nuclear ncRNAs identified in this study were higher than the average expression levels of lincRNAs (data not shown), perhaps reflecting their importance in biological processes.

Previous studies have frequently suggested tissue-specific expressions of long ncRNAs [34], [35]. In contrast, most of the long ncRNAs functionally characterized in this study are ubiquitously expressed. We selected long ncRNA candidates from the top 180 clusters containing the highest number of cDNA entries because these clusters are supposedly highly expressed in the cells. Ubiquitously expressed long ncRNAs might be preferentially selected using this criterion, because these transcripts tend to be highly represented in the database. For this reason, the majority of the long ncRNAs identified in this study are probably ubiquitously expressed.

HeLa cells are transformed by expressing the HPV E6 oncoprotein, and their transcriptional status is different from other cell lines that do not express the HPV E6 oncoprotein, such as MCF-7. The expression patterns of tissue-specific transcriptional factors of HeLa cells are different from those in MCF-7; HeLa cells and MCF-7 cells are derived from the cervical squamous cell cacrcinoma and the breast ductal carcinoma, respectively. We suspect that the cell type-dependent differential transcriptional status must influence the anticancer agent-mediated alteration of long ncRNA expression in the cells.

We initially selected ncRNA candidates by following the widely accepted ncRNA criteria: the predicted open reading frame should be less than 300 nucleotides (100 amino acids) and any Kozak sequence around the first ATG (expected initial codon) should be of low potential. However, polysome profiling using the cytosolic fraction (data not shown) revealed that four non-NMD targeted ncRNA candidates (4/29 = ∼14%) were predicted to be loaded onto ribosomes; these may be mRNAs encoding small proteins of less than 100 amino acids (micro-proteins). This observation predicts that a substantial proportion (approximately 14%) of previously annotated ncRNAs may in fact be protein-coding. Indeed, this has already been demonstrated for several ncRNAs [27], [48]–[53]. Accordingly, a bioinformatic survey of mouse cDNAs estimated the presence of approximately 1000 new genes encoding micro-proteins [54].

In summary, the novel nuclear long ncRNAs reported in this study represent an important early step in appreciating the significance of nuclear long ncRNAs in the genetic regulation of cellular stress responses. Moreover, we have identified a number of ncRNAs that respond to cellular stress, making them worthy of further study. Although the specific functions of the identified nuclear long ncRNAs remain unknown, we believe that this class of molecule will help to bridge the knowledge gap between digital genomic information and cellular function.

## Supporting Information

Figure S1 **Secondary structure prediction.** Secondary structures were predicted by mfold (http://mfold.rit.albany.edu/cgi-bin/view-folds.cgi).
(TIF)

Figure S2 **Sequence conservation across mammalian species.** Sequence conservation of UT43 (A) or UT36 (B) across 18 mammalians analyzed by UCSC genome browser. UT43 is a representative of low level of sequence conservation across mammalian species. UT36 is a representative of low level of sequence conservation.
(TIF)

Figure S3 **Tissue-distribution of ncRNAs.** The relative abundance of the indicated nuclear long ncRNAs among seven tissues and HeLa Tet-off (TO) cells was examined by qRT-PCR. The abundance in each tissue was normalized to that of *GAPDH*. (a) HeLa TO, (b) brain cortex, (c) cerebellum, (d) stomach, (e) pancreas, (f) thyroid gland, (g) lung, and (h) and heart. Error bars show the experimental error of two experiments. (A) An ncRNA expressed in several tissues. (B) Ubiquitously expressed ncRNAs.
(TIF)

Figure S4 **The expression patterns of long ncRNAs using RNA seq data from the Illumina Human BodyMap 2.0 project.** The expression profiles of long ncRNAs in the brain. The block shows how many reads aligned across an exon-exon junction. The height indicates the expression level of a transcript.
(TIF)

Table S1 **Oligonucleotides used for qRT-PCR.** All the primers sequences used in the text were listed in the table.
(XLS)

Table S2 **3,180 clusters as candidates likely to encode ncRNAs.** All the candidates likely to encode ncRNAs mentioned in the article were listed in the table.
(XLS)

Table S3 **Determination of NMD-target genes.** This table lists relative RNA level of long ncRNA candidates in UPF1 knock down cells and control cells.
(XLS)

Table S4 **The nuclear/cytoplasmic (N/C) ratio of long ncRNA candidates.** This table lists nuclear/cytoplasmic (N/C) ratio of ncRNA candidates.
(XLS)

## References

[1] Mattick JS (2004). RNA regulation: a new genetics?. Nat Rev Genet.

[2] Mattick JS (2011). Genome-sequencing anniversary. The genomic foundation is shifting.. Science.

[3] Bertone P, Stolc V, Royce TE, Rozowsky JS, Urban AE (2004). Global identification of human transcribed sequences with genome tiling arrays.. Science.

[4] Wilhelm BT, Marguerat S, Watt S, Schubert F, Wood V (2008). Dynamic repertoire of a eukaryotic transcriptome surveyed at single-nucleotide resolution.. Nature.

[5] Nagalakshmi U, Wang Z, Waern K, Shou C, Raha D (2008). The transcriptional landscape of the yeast genome defined by RNA sequencing.. Science.

[6] Birney E, Stamatoyannopoulos JA, Dutta A, Guigo R, Gingeras TR (2007). Identification and analysis of functional elements in 1% of the human genome by the ENCODE pilot project.. Nature.

[7] Brosnan CA, Voinnet O (2009). The long and the short of noncoding RNAs.. Curr Opin Cell Biol.

[8] Erwin JA, Lee JT (2008). New twists in X-chromosome inactivation. Curr Opin Cell Biol..

[9] Heard E, Disteche CM (2006). Dosage compensation in mammals: fine-tuning the expression of the X chromosome.. Genes Dev.

[10] Payer B, Lee JT (2008). X chromosome dosage compensation: how mammals keep the balance.. Annu Rev Genet.

[11] Chen LL, Carmichael GG (2009). Altered nuclear retention of mRNAs containing inverted repeats in human embryonic stem cells: functional role of a nuclear noncoding RNA.. Mol Cell.

[12] Clemson CM, Hutchinson JN, Sara SA, Ensminger AW, Fox AH (2009). An architectural role for a nuclear noncoding RNA: NEAT1 RNA is essential for the structure of paraspeckles.. Mol Cell.

[13] Sasaki YT, Ideue T, Sano M, Mituyama T, Hirose T (2009). MENepsilon/beta noncoding RNAs are essential for structural integrity of nuclear paraspeckles.. Proc Natl Acad Sci.

[14] Sunwoo H, Dinger ME, Wilusz JE, Amaral PP, Mattick JS (2009). MEN epsilon/beta nuclear-retained non-coding RNAs are up-regulated upon muscle differentiation and are essential components of paraspeckles.. Genome Res.

[15] Tano K, Mizuno R, Okada T, Rakwal R, Shibato J (2010). MALAT-1 enhances cell motility of lung adenocarcinoma cells by influencing the expression of motility-related genes.. FEBS Lett.

[16] Tripathi V, Ellis JD, Shen Z, Song DY, Pan Q (2010). A long nuclear-retained non-coding RNA regulates synaptogenesis by modulating gene expression.. EMBO J.

[17] Yu W, Gius D, Onyango P, Muldoon-Jacobs K, Karp J (2008). Epigenetic silencing of tumour suppressor gene p15 by its antisense RNA. Nature..

[18] Kotake Y, Nakagawa T, Kitagawa K, Suzuki S, Liu N (2011). Long non-coding RNA ANRIL is required for the PRC2 recruitment to and silencing of p15(INK4B) tumor suppressor gene.. Oncogene.

[19] Yap KL, Li S, Muñoz-Cabello AM, Raguz S, Zeng L (2010). Molecular interplay of the noncoding RNA ANRIL and methylated histone H3 lysine 27 by polycomb CBX7 in transcriptional silencing of INK4a.. Mol.

[20] Pandey RR, Mondal T, Mohammad F, Enroth S, Redrup L (2008). Kcnq1ot1 antisense noncoding RNA mediates lineage-specific transcriptional silencing through chromatin-level regulation.. Mol Cell.

[21] Rinn JL, Kertesz M, Wang JK, Squazzo SL, Xu X (2007). Functional demarcation of active and silent chromatin domains in human HOX loci by noncoding RNAs.. Cell.

[22] Tsai MC, Manor O, Wan Y, Mosammaparast N, Wang JK (2010). Long noncoding RNA as modular scaffold of histone modification complexes.. Science.

[23] Kültz D (2005). Molecular and evolutionary basis of the cellular stress response.. Annu Rev Physiol.

[24] Jolly C, Lakhotia SC (2006). Human sat III and Drosophila hsr omega transcripts: a common paradigm for regulation of nuclear RNA processing in stressed cells.. Nucleic Acids Res.

[25] Jolly C, Metz A, Govin J, Vigneron M, Turner BM (2004). Stress-induced transcription of satellite III repeats.. J Cell Biol.

[26] Rizzi N, Denegri M, Chiodi I, Corioni M, Valgardsdottir R (2004). Transcriptional activation of a constitutive heterochromatic domain of the human genome in response to heat shock.. Mol Biol Cell.

[27] Faghihi MA, Modarresi F, Khalil AM, Wood DE, Sahagan BG (2008). Expression of a noncoding RNA is elevated in Alzheimer's disease and drives rapid feed-forward regulation of beta-secretase.. Nat Med.

[28] Huarte M, Guttman M, Feldser D, Garber M, Koziol MJ (2010). A large intergenic noncoding RNA induced by p53 mediates global gene repression in the p53 response.. Cell.

[29] Sonkoly E, Bata-Csorgo Z, Pivarcsi A, Polyanka H, Kenderessy-Szabo A (2005). Identification and characterization of a novel, psoriasis susceptibility-related noncoding RNA gene, PRINS.. J Biol Chem.

[30] Wakamatsu A, Kimura K, Yamamoto J, Nishikawa T, Nomura N (2009). Identification and functional analyses of 11,769 full-length human cDNAs focused on alternative splicing.. DNA Res.

[31] Baker KE, Parker R (2004). Nonsense-mediated mRNA decay: terminating erroneous gene expression.. Curr Opin Cell.

[32] He F, Li X, Spatrick P, Casillo R, Dong S (2003). Genome-wide analysis of mRNAs regulated by the nonsense-mediated and 5′ to 3′ mRNA decay pathways in yeast.. Mol.

[33] Cheng J, Kapranov P, Drenkow J, Dike S, Brubaker S (2005). Transcriptional maps of 10 human chromosomes at 5-nucleotide resolution.. Science.

[34] Sasaki YT, Sano M, Ideue T, Kin T, Asai K (2007). Identification and characterization of human non-coding RNAs with tissue-specific expression.. Biochem Biophys Res Commun.

[35] Kikuchi K, Fukuda M, Ito T, Inoue M, Yokoi T (2009). Transcripts of unknown function in multiple-signaling pathways involved in human stem cell differentiation.. Nucleic Acids Res.

[36] Ebisuya M, Yamamoto T, Nakajima M, Nishida E (2008). Ripples from neighbouring transcription.. Nat Cell Biol.

[37] Leung AK, Sharp PA (2010). MicroRNA functions in stress responses.. Mol Cell.

[38] Quiñones A, Rainov NG (2001). Identification of genotoxic stress in human cells by fluorescent monitoring of p53 expression. Mutat Res..

[39] Quiles JL, Huertas JR, Battino M, Mataix J, Ramirez-Tortosa MC (2002). Antioxidant nutrients and adriamycin toxicity. Toxicology..

[40] Rajski SR, Williams RM (1998). DNA Cross-Linking Agents as Antitumor Drugs. Chem Rev..

[41] Seila AC, Calabrese JM, Levine SS, Yeo GW, Rahl PB (2008). Divergent transcription from active promoters.. Science.

[42] Affymetrix/Cold Spring Harbor Laboratory ENCODE Transcriptome Project (2009). Post-transcriptional processing generates a diversity of 5′-modified long and short RNAs.. Nature.

[43] Preker P, Nielsen J, Kammler S, Lykke-Andersen S, Christensen MS (2008). RNA exosome depletion reveals transcription upstream of active human promoters.. Science.

[44] Wyers F, Rougemaille M, Badis G, Rousselle JC, Dufour ME (2005). Cryptic pol II transcripts are degraded by a nuclear quality control pathway involving a new poly(A) polymerase.. Cell.

[45] Guttman M, Donaghey J, Carey BW, Garber M, Grenier JK (2011). lincRNAs act in the circuitry controlling pluripotency and differentiation.. Nature.

[46] Lindblad-Toh K, Garber M, Zuk O, Lin MF, Parker BJ (2011). A high-resolution map of human evolutionary constraint using 29 mammals.. Nature.

[47] Cabili MN, Trapnell C, Goff L, Koziol M, Tazon-Vega B (2011). Integrative annotation of human large intergenic noncoding RNAs reveals global properties and specific subclasses.. Genes Dev 15:.

[48] Kondo T, Hashimoto Y, Kato K, Inagaki S, Hayashi S (2007). Small peptide regulators of actin-based cell morphogenesis encoded by a polycistronic mRNA.Nat. Cell Biol..

[49] Hanyu-Nakamura K, Sonobe-Nojima H, Tanigawa A, Lasko P, Nakamura A (2008). Drosophila Pgc protein inhibits P-TEFb recruitment to chromatin in primordial germ cells.. Nature.

[50] Emberley E, Huang GJ, Hamedani MK, Czosnek A, Ali D (2003). Identification of new human coding steroid receptor RNA activator isoforms.. Biochem Biophys Res Commun.

[51] Wadler CS, Vanderpool CK (2007). A dual function for a bacterial small RNA: SgrS performs base pairing-dependent regulation and encodes a functional polypeptide.. Proc Natl Acad Sci.

[52] Balaban N, Novick RP (1995). Translation of RNAIII, the Staphylococcus aureus agr regulatory RNA molecule, can be activated by a 3′-end deletion.. FEMS Microbiol Lett.

[53] van de Sande K, Pawlowski K, Czaja I, Wieneke U, Schell J (1996). Modification of phytohormone response by a peptide encoded by ENOD40 of legumes and a nonlegume.. Science.

[54] Frith MC, Forrest AR, Nourbakhsh E, Pang KC, Kai C (2006). The abundance of short proteins in the mammalian proteome.. PLoS Genet.

