# Supplementary figures and images for: Identification and Characterization of Novel Genotoxic Stress-Inducible Nuclear Long Noncoding RNAs in Mammalian Cells
Source: PLoS One. 2012 Apr 19;7(4):e34949. doi: 10.1371/journal.pone.0034949 (PMC3330809; doi:10.1371/journal.pone.0034949)

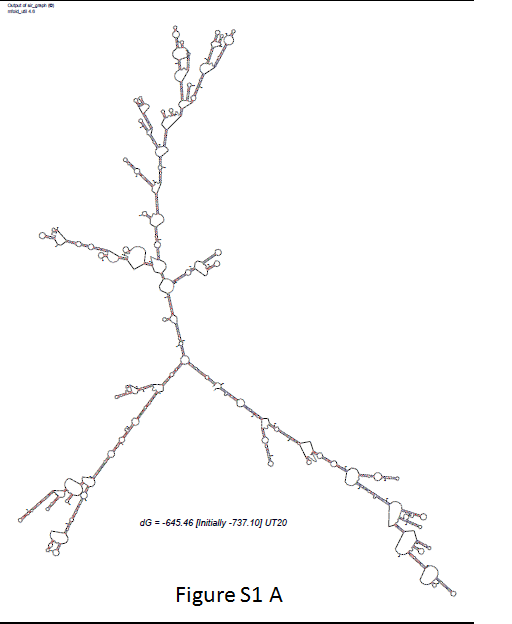

Supplement: Figure S1 — Secondary structure prediction. Secondary structures were predicted by mfold (http://mfold.rit.albany.edu/cgi-bin/view-folds.cgi). (TIF) [file pone.0034949.s001.tif]

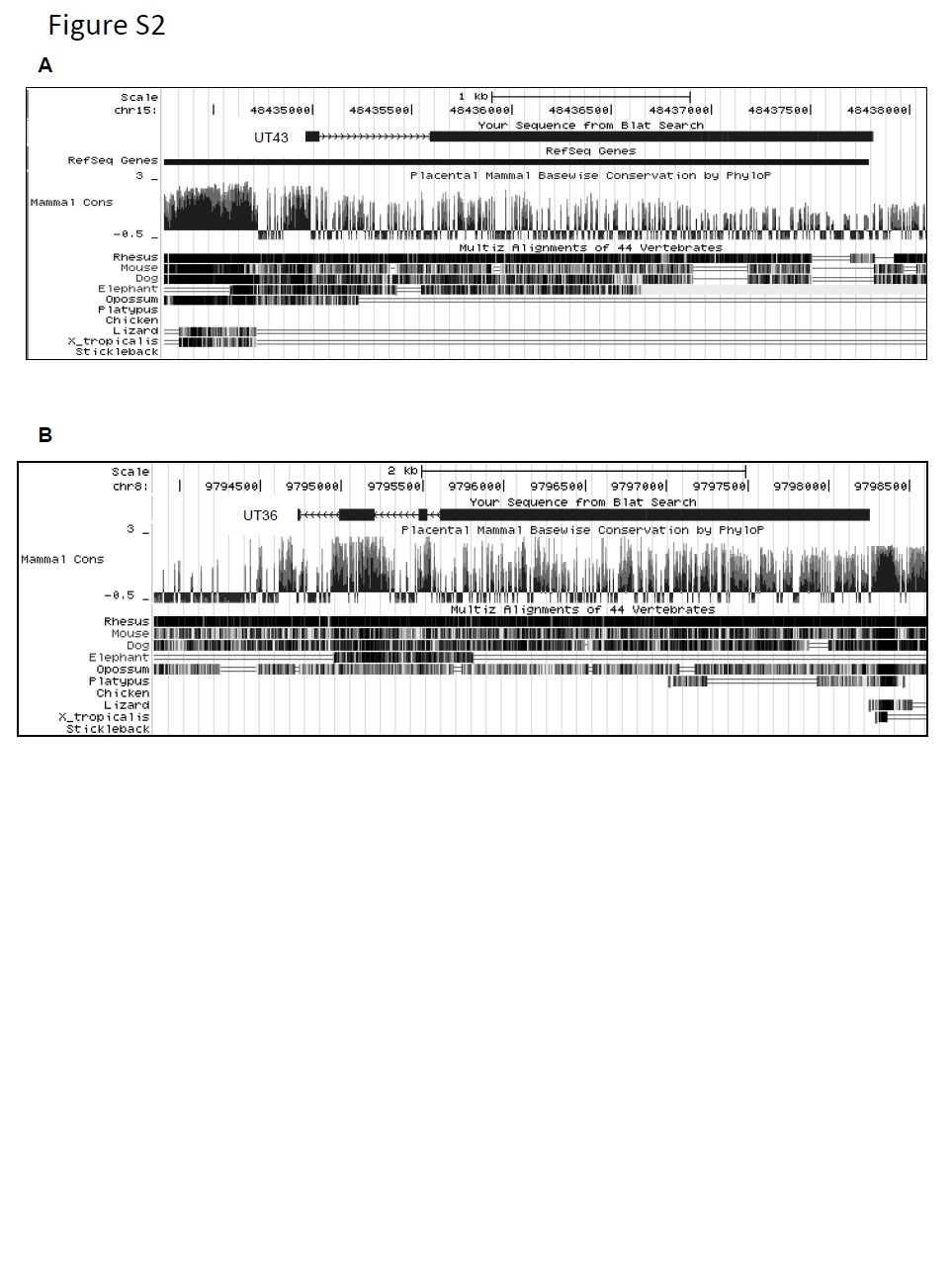

Supplement: Figure S2 — Sequence conservation across mammalian species. Sequence conservation of UT43 (A) or UT36 (B) across 18 mammalians analyzed by UCSC genome browser. UT43 is a representative of low level of sequence conservation across mammalian species. UT36 is a representative of low level of sequence conservation. (TIF) [file pone.0034949.s002.tif]

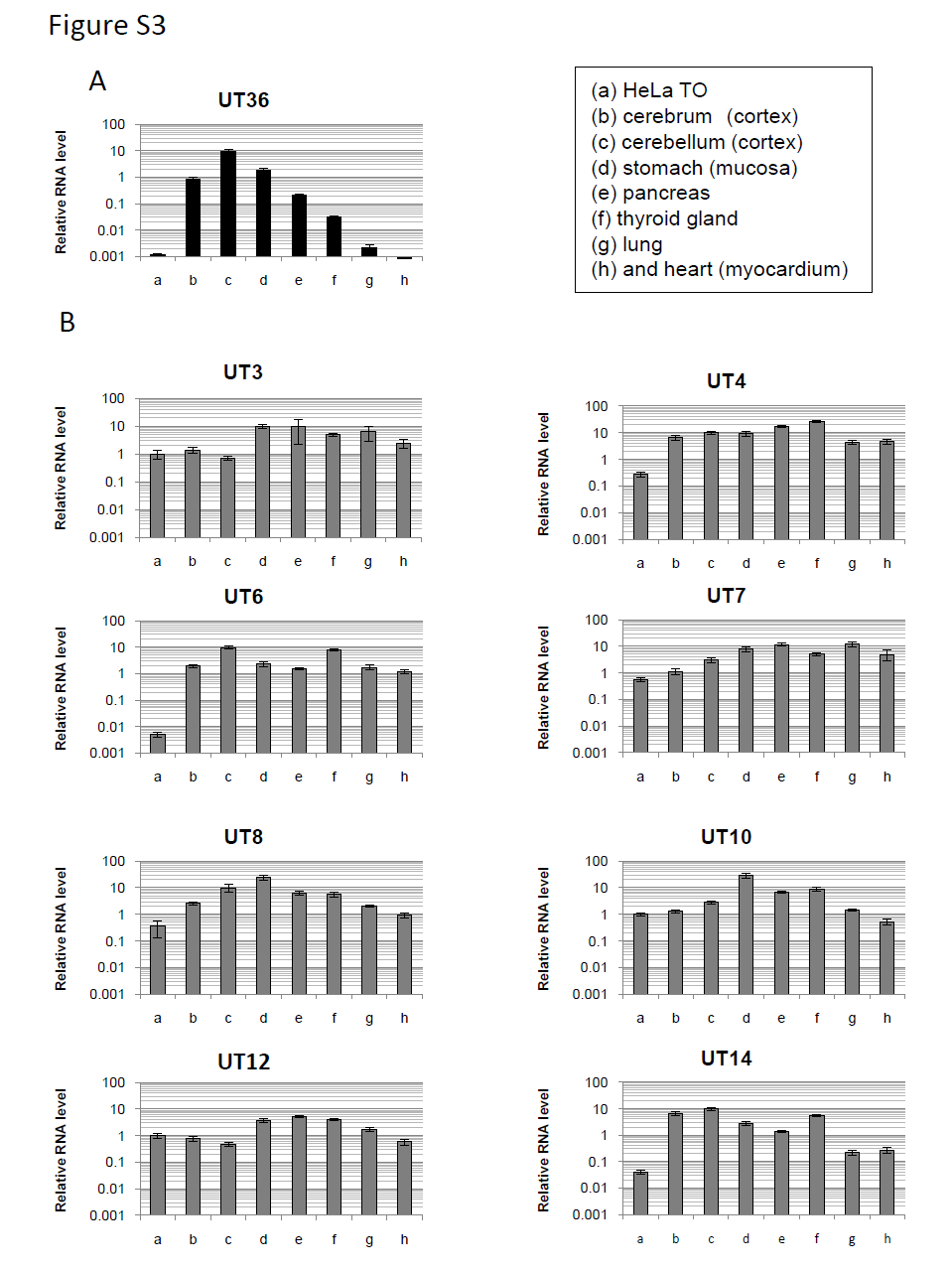

Supplement: Figure S3 — Tissue-distribution of ncRNAs. The relative abundance of the indicated nuclear long ncRNAs among seven tissues and HeLa Tet-off (TO) cells was examined by qRT-PCR. The abundance in each tissue was normalized to that of GAPDH. (a) HeLa TO, (b) brain cortex, (c) cerebellum, (d) stomach, (e) pancreas, (f) thyroid gland, (g) lung, and (h) and heart. Error bars show the experimental error of two experiments. (A) An ncRNA expressed in several tissues. (B) Ubiquitously expressed ncRNAs. (TIF) [file pone.0034949.s003.tif]

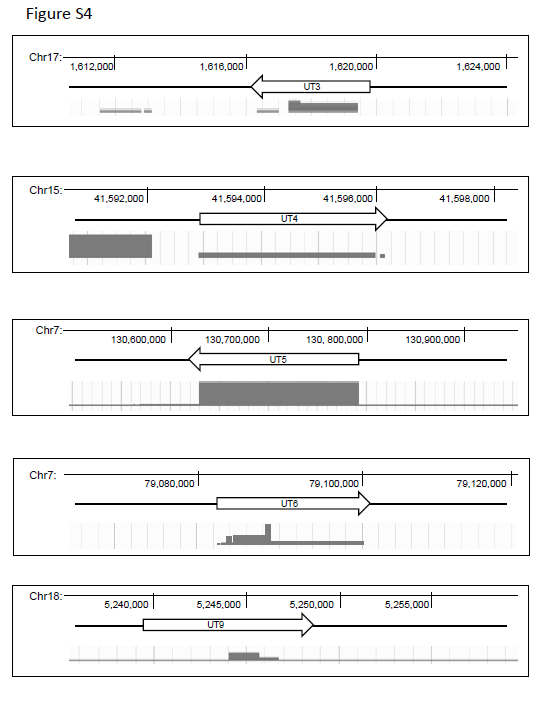

Supplement: Figure S4 — The expression patterns of long ncRNAs using RNA seq data from the Illumina Human BodyMap 2.0 project. The expression profiles of long ncRNAs in the brain. The block shows how many reads aligned across an exon-exon junction. The height indicates the expression level of a transcript. (TIF) [file pone.0034949.s004.tif]
